# Optimal Vase Solution for *Gerbera hybrida* Cut Flower Keeping Fresh by Activating SA and Cytokinin Signaling and Scavenging Reactive Oxygen Species

**DOI:** 10.3390/biology14010018

**Published:** 2024-12-28

**Authors:** Chaoshui Xia, Yiyang Cao, Weixin Gan, Huifeng Lin, Huayang Li, Fazhuang Lin, Zhenhong Lu, Weiting Chen

**Affiliations:** 1 Sanming Academy of Agricultural Sciences, Sanming 365509, China; xiachaoshui_smnks@163.com (C.X.); caoyiyang_smnks@163.com (Y.C.); ganweixin_smnks@163.com (W.G.); linhuifeng_smnks@163.com (H.L.); 2 Fujian Key Laboratory of Crop Genetic Improvement and Innovative Utilization for Mountain Area, Sanming 365509, China; 3 Horticulture College, Fujian Agriculture and Forestry University, Fuzhou 350002, China; 4 Institute of Vegetables and Flowers, Chinese Academy of Agricultural Sciences, Beijing 100081, China; lihuayang@caas.cn; 5 Yunnan Academy of Agricultural Sciences, Kunming 650205, China; luzhenhong9836@163.com

**Keywords:** *Gerbera hybrida*, preservation, response surface, antioxidant activity, transcriptome

## Abstract

Gerbera (*Gerbera hybrida*) is a popular cut flower on the market, so extending its vase life (VL) is an important goal in the horticultural industry. The aim of this study was to improve the freshness of gerbera cut flowers through the optimal solution (OS) and to analyze its preservation mechanism. We used chitosan (COS), calcium chloride (CaCl_2_), and citric acid (CA) as the main ingredients of the vase solution and determined the OS ratio of 104 mg/L of COS, 92 mg/L of CA, and 93 mg/L of CaCl_2_ using the Box–Behnken design-response surface method (BBD-RSM). Gerbera preservation results showed that the VL of the OS was 14.5 days, which was significantly longer than that of flowers maintained in the Basic Vase Solution (BVS) and the Commercial Formulation (CF) and was highly consistent with the theoretical VL of 14.57 d. Transcriptome analysis indicated that the OS might extend VL by regulating phytohormone signaling pathways, such as cytokinin and salicylic acid signaling. The qRT-PCR analysis of key candidate genes supported these findings, with significant upregulation observed in genes related to cytokinin synthesis (e.g., *GhIPT1* and *GhIPT9*), salicylic acid signaling related to pathogen defense (e.g., *GhTGA1*, *GhTGA4*, *GhNPR1*, and *GhRBOHA*), and plant wax synthesis and stress response (e.g., *GhKCS5*, *GhCUT1*, and *GhKCS6*). Further, transcriptome GO-enrichment and physiological analysis showed that the OS might extend VL of Gerbera cut flowers by scavenging reactive oxygen species, including by activating the expression of genes related to oxidoreductase activity and the activities of antioxidant-system-related enzymes catalase (CAT), peroxidase (POD), ascorbate peroxidase (APX), and superoxide dismutase (SOD), while decreasing the malondialdehyde (MDA) content. These results provide valuable insights into the mechanisms underlying the extended VL of gerbera cut flowers and offer a foundation for developing more effective preservation techniques.

## Simple Summary

Choosing a suitable preservative can significantly prolong the life of fresh-cut flowers. We optimized the preservative formula of *Gerbera hybrida* and made it last longer. On this basis, we found that this improved preservative formula works by activating cytokinin and salicylic acid signaling, and this preservative formula is also helpful to improve the antioxidant activity of *Gerbera hybrida*. Our findings provide a theoretical basis for the optimization of *Gerbera hybrida* preservatives in the future.

## 1. Introduction

*Gerbera hybrida*, a member of the Asteraceae family, is highly valued in the global cut flower market for its diverse array of colors and shapes [1]. However, a significant challenge faced by consumers is the limited vase life (VL) of these flowers under standard room conditions [2]. This shortened VL of gerbera cut flowers is primarily due to scape bending, even when the ray petals remain unwilted [3]. Previous studies have identified that scape bending predominantly occurs in the neck region of the gerbera stem, especially the 12 cm below the floral head [4]. Despite numerous reports on the physiological processes involved in petal senescence in gerbera cut flowers, there is a notable lack of understanding regarding the molecular aspects of chrysanthemum petal senescence. Consequently, it is imperative for the horticulture industry to focus on enhancing the VL and quality of cut flowers by regulating postharvest gerbera and addressing these challenges. Improving VL and storage characteristics has become a primary breeding objective for gerbera, as these factors significantly impact consumer satisfaction and the global transportability of the flowers [5].

Numerous studies have explored the preservation of gerbera cut flowers, primarily categorized into physical and chemical methods. Chemical preservation typically involves the direct application of composite preservatives to prolong freshness [6]. Among the commonly recognized economical, effective, and environmentally friendly components of flower preservatives are chitosan (COS), calcium chloride (CaCl_2_), and citric acid (CA). COS, a high-molecular-weight polymer renowned for its exceptional antibacterial properties [6], is non-toxic and completely safe for human use. This versatile compound is widely applied in the preservation of fruits, vegetables, and flowers. CaCl_2_ has been shown to effectively maintain the beauty and vitality of gerbera cut flowers by inhibiting stem bending, a key factor limiting VL. Application methods such as spraying, dipping, or injecting with CaCl_2_ have been proven successful in prolonging the longevity of these blossoms [7]. CA, a natural organic acid with widespread use in browning inhibition, also contributes to floral preservation [8]. However, the preservative formulations used in previous studies on *Gerbera hybrida* often pose higher risks of harm due to the use of single components or the need for specific conditions to achieve preservation effects [8]. Many of these formulations fail to provide consistent, significant results. Therefore, optimizing preservative formulas with economic and environmentally friendly components remains essential for improving the VL and quality of gerbera cut flowers.

The senescence and loss of ornamental value of cut flowers are closely linked to the production of reactive oxygen species (ROS) [9], which induce oxidative stress and lead to senescence by degrading proteins, lipids, and nucleic acids. Plants have an antioxidant protection system to neutralize harmful free radicals [10]. This system includes non-enzymatic antioxidants such as ascorbic acid, α-tocopherol, glutathione, phenolic compounds, and flavonoids, as well as a series of enzyme systems, including catalase (CAT), superoxide dismutase (SOD), peroxidase (POD), and ascorbate peroxidase (APX) [11]. Fresh-cut flower preservatives can extend VL by enhancing these antioxidant defense mechanisms. For instance, the application of exogenous sodium nitroprusside (SNP) and gibberellic acid (GA3) to dahlia cut flowers has been shown to improve postharvest quality by modulating antioxidant activities, including CAT, SOD, POD, and malondialdehyde (MDA) [12]. A similar phenomenon was observed in carnation-cut flowers, where the application of preservatives increased the membrane stability index (MSI) and the antioxidant activities of CAT and POD, ultimately leading to an extended VL [13]. In gerbera cut flowers, borage leaf extract extended VL by mitigating oxidative stress, reducing lipid peroxidation such as MDA, and alleviating water stress during senescence [14]. The enhancement of the antioxidant system is often a key indicator used to evaluate the effectiveness of flower preservatives, whereas the detailed mechanism for optimized preservative formulations and this function in affecting the antioxidant system for *Gerbera hybrida* are limited.

Numerous studies have explored the physiological processes associated with petal senescence in gerbera cut flowers, identifying stem bending as a primary factor limiting their VL [12]. The introduction of bacteria into the vase water of freshly harvested gerbera cut flowers rapidly induces stem bending, as bacterial blockage disrupts water uptake [12]. Stem bending is usually accompanied by the presence of a thin sclerenchyma cylinder approximately 20–30 cm below the flower head and low stem lignin contents [12]. Research highlights the critical role of hormone balance in regulating the senescence of fresh-cut flowers [15]. For instance, brassinosteroids (BRs) have been shown to influence various physiological and developmental processes in gerbera, including cell division and elongation, leaf expansion, vascular differentiation, and stomatal development. BRs also participate in multiple hormone signaling and biosynthetic pathways [16,17]. Despite these advances, few studies have addressed the molecular mechanisms underlying the longevity of gerbera cut flowers. The transcriptome, a valuable tool for analyzing large-scale transcriptional regulatory events, has been widely used to study gene expression and the pathways involved in petal senescence [18]. However, little attention has been given to understanding the molecular mechanisms by which high-quality preservatives delay petal senescence.

In this study, we initially screened COS, CaCl_2_, and CA as the primary components of the preservative for gerbera cut flowers through a one-way ANOVA test. Subsequently, an optimized preservative formulation was developed using the Box–Behnken design method, a statistical technique that facilitates the establishment of mathematical models between multiple variables, particularly effective in experiments involving three or more independent variables. This method is based on the concept of response surface design, enabling us to explore the interactive effects between factors efficiently. Furthermore, we employed response surface analysis (RSA), a statistical and mathematical approach that helps optimize the impact of multiple variables in engineering and scientific problems [19]. Through RSA, we were able to construct a quadratic polynomial regression model to fit the relationship between COS, CA, and CaCl_2_ and their influence on VL. This model developed using Design-Expert 12 Trial software(v12.0.3.0), captures the linear and non-linear effects of the factors as well as their interactions. The high significance of the model was confirmed through analysis of variance (ANOVA) and a test of the model’s validity, with a *p*-value less than 0.01, indicating a very significant statistical model (*p* < 0.01). In summary, our research not only identified key factors affecting the VL of gerbera cut flowers but also optimized the preservative formulation and established a predictive model to guide future experiments and applications, which is of significant practical importance for enhancing the longevity of cut flowers.optimal solution (OS) can improve the freshness of gerbera cut flowers and prolong their VL. We optimized the OS of gerbera, and the optimized formulation has more advantages over the previous one. In addition, we also used the transcriptome to preliminary analyze the effect of the OS on the gene expression of gerbera and found that it can prolong the VL by activating the salicylic acid (SA) and cytokinin signaling pathways and scavenging reactive oxygen species (ROS). Our findings provide new insights into the preservation of gerbera cut flowers and lay the foundation for the development of more efficient preservation techniques.

## 2. Materials and Methods

### 2.1. Plant Materials and Growth Conditions

The trial was carried out in November 2022 at the Fujian Key Laboratory of Crop Genetic Improvement and Innovative Utilization for Mountain Areas, Sanming Academy of Agricultural Sciences. The tested gerbera variety, ‘Minghui Zixia’, was collected from the National Germplasm Resources Bank of the Sanming Academy of Agricultural Sciences. This variety, independently developed by the Sanming Academy of Agricultural Sciences, was the first gerbera variety in Fujian Province to receive a new plant variety certificate from the Ministry of Agriculture and Rural Affairs of China. This variety has the advantages of rare flower color, high yield, strong adaptability, resistance to powdery mildew and fusarium wilt, good marketability, etc. This variety is prone to a short VL after harvesting, which reduces the economic benefits. The most commonly used fresh-cut flower commodity preservative in the Chinese market, Chrysal Clear Universal Flower Food, was used as a Commercial Formulation (CF). Ambient conditions: Temp: 25 °C, Light 3LS, RH: 60% in an artificial climate chamber. Solution supplement: During the experiment, in order to maintain the freshness and effectiveness of the bottled solution, we regularly replaced the bottled solution. The specific supplement frequency and method depend on the consumption rate of the solution and the actual demand of the cut flower. We ensure that each time the solution is replaced, a freshly prepared solution is used to provide the necessary nutrition and fresh-keeping effects.

### 2.2. Optimization of Freshness-Preserving Agent Formula via the Response Surface Method

In our experiment, we initially screened chemical preservatives based on economic efficiency to assess their impact on the VL of gerbera cut flowers. Each treatment group utilized six flowers. Basic Vase Solution (BVS) consisting of 3% sucrose and 250 mg/L 8-hydroxyquinoline (8-HQ), we tested additional freshness-preserving agents. In all our experiments, a base solution containing sucrose and 8-hydroxyquinoline (8-HQ) was employed. The agents included an antimicrobial agent (A: tea polyphenol (TP) or B: chitosan (COS)), an organic acid (C: citric acid (CA) or D: salicylic acid(SA)), and an inorganic salt (E: calcium chloride (CaCl_2_) or F: sodium chloride(NaCl)), which were added to evaluate their efficacy as freshness-preserving agents for cut flowers (Table S1). The experiment was conducted with three replicates, and the concentrations of the selected chemical reagents were 50 mg/L, 100 mg/L, and 150 mg/L. Data statistics were performed using the Student’s *t*-test, where ns denotes not significant, * indicates significant (*p* < 0.05), and ** indicates highly significant (*p* < 0.01). A randomized block group design was used, with the BVS as the control group. The maximum flower size and VL were recorded for each treatment.

The Design-Expert 12 Trial software provided the function for Box–Behnken Design (BBD) for optimizing experimental conditions. Using the single-factor test, the optimal types of antimicrobial agents, organic acids, and inorganic salts were selected as independent variables for further optimization using response surface methodology. Taking the VL (d) of different concentrations of antimicrobial agents as the response value, the response surface design was carried out by the Box–Behnken central experimental design principle of Design-Expert 12 Trial software. The optimal formula was analyzed by the response surface regression equation, which was applied to OS of gerbera cut flowers.

### 2.3. Morphological Observation and Physiological and Biochemical Assays

OS was applied to a preservation experiment for gerbera cut flowers, with the basic vase solution as the control and a commercially available preservative, Chrysal Clear Universal Flower Food, as the CF. The experiment measured the maximum flower diameter and recorded the VL of the cut flowers.

For enzyme activity assays, petal powder (0.1 g) was extracted using 100 mM cold sodium phosphate buffer (pH 6.8) supplemented with 1 mM phenylmethylsulfonyl fluoride (Sigma-Aldrich, St. Louis, USA). The enzyme activities of ascorbate peroxidase (EC 1.11.1.11), catalase (EC 1.11.1.6), and peroxidase (EC 1.11.1.7) were determined following previously established methods [20,21,22]. Superoxide dismutase (EC 1.15.1.1) activity was measured using a SOD assay kit (Solarbio, Beijing, China), and absorbance values were recorded with an Infinite M200 plate reader (Tecan Group Ltd, Männedorf, Switzerland) according to the manufacturer’s instructions.

To determine malondialdehyde (MDA) levels, petals were ground in liquid nitrogen, and the resulting petal powder was homogenized in 1× phosphate-buffered saline (PBS, pH 7.4). MDA content was measured using an MDA assay kit (Solarbio, Beijing, China), with absorbance recorded at 450, 532, and 600 nm in accordance with the manufacturer’s protocol.

### 2.4. RNA Extraction and RNA-Seq Analysis

The ligation products were size-selected via agarose gel electrophoresis, PCR-amplified, and sequenced using Illumina novaseq 6000 from Genedenovo Biotechnology Co. (Guangzhou, China). Total RNA was extracted using the Trizol method (Invitrogen, Carlsbad, CA, USA). RNA concentration and purity were measured using a NanoDrop 2000 (Thermo Fisher Scientific, Wilmington, DE, USA). RNA integrity was assessed using an RNA Nano 6000 test kit from the Agilent Bioanalyzer 2100 system (Agilent Technologies, Santa Clara, CA, USA). Two cDNA libraries were constructed for the gerbera materials treated with the BVS: the control (BVS1, BVS2, and BVS3, 3 replicates) and the 6-day preservative OS group (OS1, OS2, and OS3, 3 replicates). RNA-Seq was performed using a de novo assembly approach. Following quality assessment, the libraries were sequenced on the Illumina HiSeq platform. High-quality reads were assembled into unigenes using Trinity [23]. The Benjamani–Hochberg method was used to calibrate the hypothesis testing probability (*p*-value) using multiple hypothesis testing and to obtain the false discovery rate (FDR). Differential expression analysis between the two groups was performed using DESeq2 [24], with an FDR < 0.01 and a fold change ≥ 2 as thresholds for significant differential expression. Additionally, the differential expression, functional annotation, and functional enrichment of genes between the 6-day BVS group and the 6-day OS group were comprehensively analyzed.

### 2.5. Reverse-Transcription Polymerase Chain Reaction (RT-PCR) and Gene Expression Analysis

Total RNA was extracted from 0.1 g of frozen petal tissue using the pine tree method [25]. Using the PrimeScript™ RT Reagent Kit with gDNA Eraser (Takara Bio Inc., Shiga, Japan), one microgram of the extracted RNA was then converted to complementary (c)DNA according to the manufacturer’s guidelines. The PCR reaction was carried out according to the previous method [26], using gene-specific primers (Table S2) to amplify genes.

The real-time quantitative (qRT)-PCR amplification protocol included an initial cycle at 95 °C for 30 s, followed by 45 cycles at 95 °C for 5 s and 60 °C for 20 s, using the ABI QuantStudio 3 system (Applied Biosystems, Carlsbad, CA, USA). Gene expression levels were quantified using the 2^−ΔΔct^ method. For normalization of gene transcript levels, the *GhActin6* gene was co-amplified as a reference gene.

### 2.6. Statistical Analysis

For response surface analysis, the Box–Behnken central experimental design principle of Design-Expert 12 Trial software was used, and the direction of the influence was determined based on the positive or negative coefficients. A quadratic polynomial regression model for VL (y) was established with chitosan (A), citric acid (B), and calcium chloride (C) as variables. The analysis of variance (ANOVA) and a test of the validity of the model were also performed using Design-Expert 12 Trial software.

All other statistical analyses were performed using R software (version 4.2.3). For pairwise comparisons, the Student’s *t*-test was used. A significance level of *p* < 0.05 was considered statistically significant. Data visualization, including volcano plots and response surface graphs, was performed using the ggplot2 package.

## 3. Results

### 3.1. Screening of Freshness Preservation Agents for Gerbera Cut Flowers

**Figure 1:**
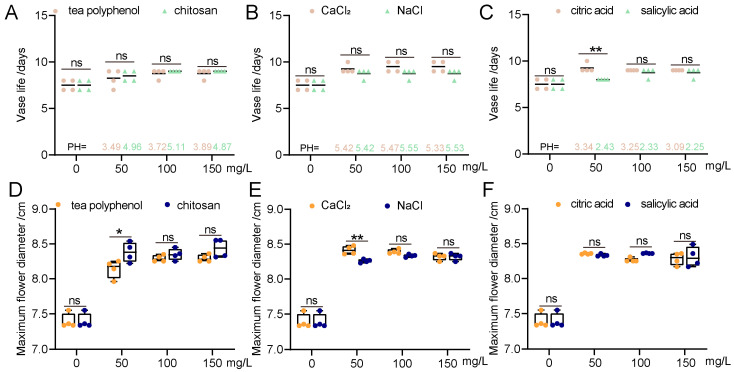
Effect of a single-factor chemical agent for freshness preservation. (**A**) Effect of antibacterial agent on VL of gerbera cut flower. (**B**) Effects of organic acids on the VL of cut gerbera flowers. (**C**) Influence of inorganic salts on VL of gerbera cut flowers. (**D**) Effect of antibacterial agent on maximum flower diameter of gerbera cut flower. (**E**) Effects of organic acids on the maximum flower diameter of cut gerbera flowers. (**F**) Influence of inorganic salts on maximum flower diameter of gerbera cut flowers. In the context of statistical analysis, “ns” stands for “not significant,” which means that the differences between the two groups have not reached a level of statistical significance. “*” denotes “significant,” and is commonly used to indicate a *p*-value less than 0.05, suggesting that the differences between the groups are statistically significant. “**” signifies “highly significant”, which typically corresponds to a *p*-value less than 0.01, indicating that the differences between the groups are highly statistically significant.

In this study, we conducted a VL test of gerbera cut flowers using various concentrations of freshness preservation agents, including TP, COS, CA, SA, CaCl_2_, and NaCl_2_ (Figure 1). After processing with these agents, no significant difference was observed for the concentration of TP with 50 mg/L or 100 mg/L and the concentration of NaCl_2_ with 50 mg/L, while all of the other treatments significantly increased flower size and prolonged the VL of gerbera cut flowers. Specifically, COS was more effective than TP in prolonging the VL, and COS performed better than TP in promoting the flower size; thus, we chose COS as the potential antimicrobial agent (Figure 1A,D). As for organic acids, both CA and SA also significantly prolonged the VL compared to the control, and CA functioned better in maintaining a larger flower diameter compared to SA (Figure 1B,E). As for the inorganic salts, both CaCl_2_ and NaCl_2_ could significantly enhance the VL and flower diameter of gerbera compared with the control group, but CaCl_2_ had a more significant effect at different concentrations (Figure 1C). Combining these results, we identified the three components, COS, CA, and CaCl_2_, as the main active ingredients of the preservative and used them as the independent variables in the subsequent response surface analyses to further optimize the vase solution.

### 3.2. Novel Vase OS Using Box–Behnken Design-Response Surface Method (BBD-RSM) Significantly Extends the VL of Gerbera Cut Flowers

We then employed the Box–Behnken central combination design to optimize the preservative formula, selecting COS, CA, and CaCl_2_ as independent variables in the response surface analysis. The formula of the combined agents was optimized via the Box–Behnken test, and experiments were conducted accordingly (Table S3). The Box–Behnken test design was fitted via a quadratic polynomial regression model in Design-Expert 12 Trial software. The model predicted VL (y) as a function of COS (A), CA (B), and CaCl_2_ (C) by evaluating the coefficients of positive and negative directions (Table S3). The regression equation for VL was derived from ANOVA and is expressed as: y = 14.48 + 0.20A − 0.57B − 0.50C − 0.83AB + 0.72AC + 0.13BC − 1.50A^2^ − 2.15B^2^ − 1.70C^2^.

**Figure 2:**
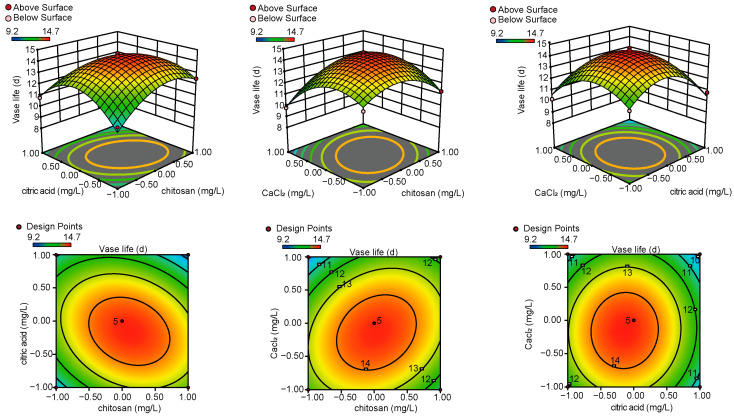
Response surface diagram (**top**) and contour plot (**bottom**) of interaction of different insurance agents on VL of gerbera cut flowers.

The analysis of variance and validity test of the regression equation showed that the model was highly significant (*p* < 0.01). The lack-of-fit *p*-value was 0.065, greater than 0.05, indicating that the model was appropriately selected and could accurately reflect the relationship between COS, CA, CaCl_2_, and VL (Table S4). The correlation coefficient (R^2^) was 0.9567, suggesting a strong fit of the model. The coefficient of variation (CV) is 3.28%, indicating that the model equation closely represents the real experimental values and can reliably predict the optimal ratio of the three components (Table S5). To illustrate the effects of two factors on VL, we first take one factor as a fixed value and analyze the influence of the other two factors on VL. Notably, there was some interaction between CA and CaCl_2_ (BC). The contour shape for CA and CaCl_2_ (AC) was round, indicating no significant interaction between them. The interactions between COS and CA (AB) and COS and CaCl_2_ (AC) were elliptical, indicating clear interactions between these variables. From the three-bit plane diagram (3DSurface), we can see that the three response surface curves are all downward concave surfaces, indicating that there is an ideal solution for the COS, CA, and CaCl_2_ concentrations in the OS group (y) (Figure 2). In order to maintain the VL, the OS conditions are as follows: COS concentration, 103.78 mg/L; CA concentration, 92.44 mg/L; and CaCl_2_ concentration, 93.17 mg/L. Theoretically, the predicted VL of the OS group (y) is 14.57 d. Considering the feasibility and convenience of the actual operation, the OS group preservative formula was revised as follows: COS 104 mg/L, CA concentration 92 mg/L, CaCl_2_ concentration 93 mg/L.

**Figure 3:**
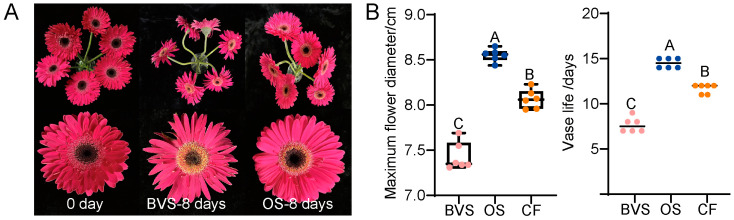
(**A**) Different stages and treatments of gerbera cut flowesr bottling state. (**B**) Effect of OS preservative on VL and maximum flower diameter of gerbera cut flowers, BVS: Basic Vase Solution; OS: optimal solution; CF: Commercial formulation.The dots in the graph represent the number of repetitions. The dots with different colors in subfigure. (**B**) represent different groups or categories of data. Different uppercase letters indicate that the differences between them are significant at the *p* < 0.01 level of significance.

In order to verify the freshness preservation effect of OS on gerbera cut flowers, OS was applied, and its freshness preservation effects were assessed using the BVS and CF after gerbera cut flowers had been in a vase for 8 days. Compared with the BVS, the state of gerbera cut flowers treated with the OS displayed superior freshness preservation (Figure 3A). The whole flower remained upright, with no bending of the stems, and the petals showed no signs of fading, wilting, or other deterioration commonly associated with the end of VL. The maximum flower diameter was measured, showing that flowers treated with the OS had a diameter of 8.56 cm, significantly larger than those in the CF and BVS groups (Figure 3B). This result indicates that the cut flowers in the OS group exhibited the best marketability. The average VL for the control group and the CF group was 7.5 and 12.5 days, respectively, while the OS group achieved an average VL of 14.5 days, significantly longer than BVS and CF groups (Figure 3B). Importantly, the actual VL of the OS group was 14.5 days, which was only 0.07 days shorter than the model’s predicted VL (14.57 d), resulting in a relative error of 0.005 (<0.05). These results demonstrate a high degree of agreement between the model predictions and the experimental results, confirming that the quadratic polynomial regression equations accurately reflect the response variables.

### 3.3. Large-Scale Expression Profile Alterations in Gerbera Cut Flowers Affected by OS

**Figure 4:**
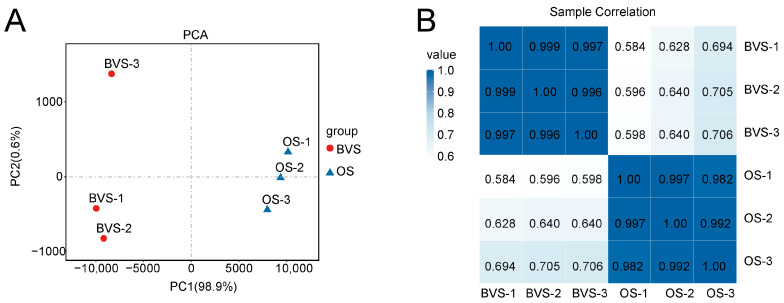
(**A**) Principal component analysis (PCA) of the samples of BVS vs. OS gerbera cut flowers. (**B**) Sample correlation heat map.

To elucidate the molecular mechanisms by which the OS extends the VL of gerbera cut flowers, we conducted transcriptome sequencing on the petals of flowers treated with the BVS and the OS after 6 days. After filtering the raw sequencing data, we obtained a total of 242,521,936 clean reads. The Q20 value of six samples was higher than 97.40%, and the Q30 value was greater than 92.98%, with a GC percentage ranging from 44.26 to 44.58%, indicating good-quality transcriptome sequencing (Table S6). The de novo analysis identified 67,821 unigenes with an N50 length of 1621 nucleotides. The expression profile of each sample was evaluated using the assembled unigenes. Subsequently, we performed principal component (PCA) and correlation analyses on the transcriptome data, and the results showed that the correlation between different group samples was low. The PCA results revealed a clear separation between the BVS and OS groups, with distinct clustering observed for each group (Figure 4A). The correlation heatmap further illustrated a low correlation between samples from different groups while demonstrating high repeatability among replicates within the same group (Figure 4B). In summary, our data suggest that the sequences were of high quality, meeting the requirements for subsequent analyses.

**Figure 5:**
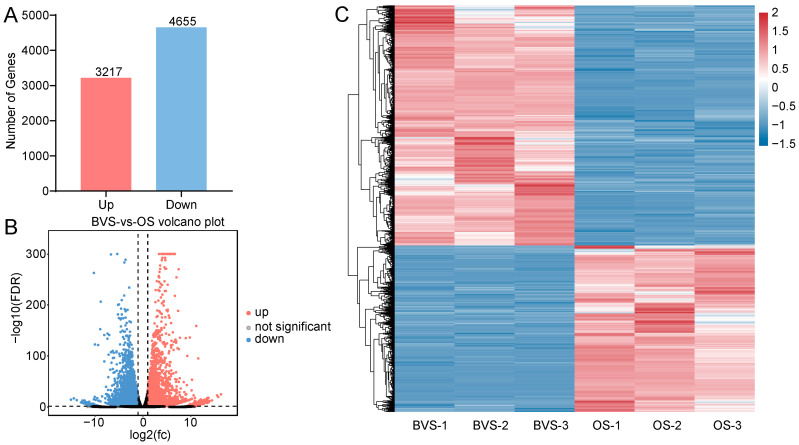
(**A**) Numbers and (**B**) volcano plot of up- and down-regulated DEGs in control (BVS) vs. OS gerbera cut flower, and (**C**) clustering heat map of DEGs in BVS vs. OS gerbera cut flower.

Compared to the control samples, the differentially expressed genes (DEGs) in the petals of gerbera cut flowers treated with OS were screened using DESeq2, resulting in 7872 DEGs. Among these DEGs, a total of 3217 genes were upregulated and 4655 genes were downregulated (Figure 5A,B). The expression patterns of those DEGs between the control group and the T group were evaluated with their FPKM values, suggesting the good reproducibility within the group (Figure 5C).

### 3.4. The OS Might Extend the VL of Gerbera Cut Flowers by Plant Signal Transduction

**Figure 6:**
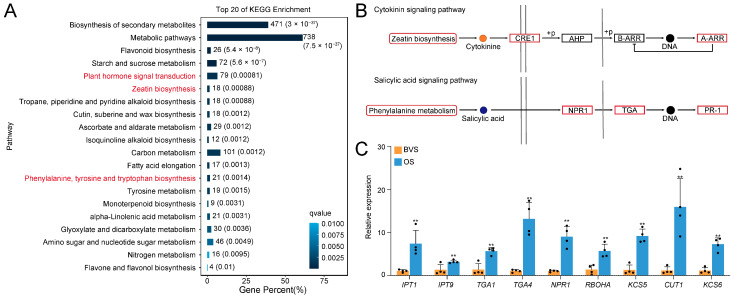
The top 20 enriched KEGG pathways for the upregulated DEGs in BVS vs. OS gerbera cut flowers. (**A**) The KEGG pathways were enriched in the upregulated DEGs. (**B**) Parts of the cytokinin and salicylic acid signaling pathways were significantly expressed under treatment (red). (**C**) Detection of key differentially expressed genes using qRT-PCR. Data in (**C**) represent the means ± SD from four independent experiments. Error bars indicate SD. “**” above the bars indicates significant differences (*p* < 0.01) calculated by Fisher’s protected *t*-test. The dots in the graph represent the number of repetitions. The pathways marked in red are Closely related to cytokinin signaling and salicylic acid signaling.

To further analyze the functions of these DEGs, we performed KEGG enrichment analysis. The results revealed that DEGs were significantly enriched in the plant hormone signaling processes. Additionally, the pathways “Zeatin biosynthesis” and “Phenylalanine, tyrosine, and tryptophan biosynthesis” were significantly enriched, both of which are closely related to cytokinin signaling and salicylic acid signaling (Figure 6A). Further analysis showed that, compared to the control group, the expression levels of genes in key components of the cytokinin and salicylic acid signaling pathways were significantly increased, suggesting that the treatment strongly activated these pathways and their associated genes (Figure 6B). To validate these findings, fluorescence qRT-PCR was conducted to examine the expression levels of key genes. These included cytokinin synthesis genes (isopentenyl transferases: *GhIPT1* and *GhIPT9*), salicylic acid signaling pathway receptors and transcription factors (TGACG motif-binding factor: *GhTGA1* and *GhTGA4*; nonexpressor of pathogenesis-related genes 1: *GhNPR1* and respiratory burst oxidase homologue A: *GhRBOHA*), as well as genes associated with disease resistance, wax biosynthesis, and stress tolerance (3-ketoacyl-CoA synthase: *GhKCS5*, *GhCUT1,* and *GhKCS6*). After treatment, the expression levels of these genes were significantly higher than those in the control samples (Figure 6C).

### 3.5. The OS May Extend the VL of Gerbera Cut Flowers by Scavenging Reactive Oxygen Species

**Figure 7:**
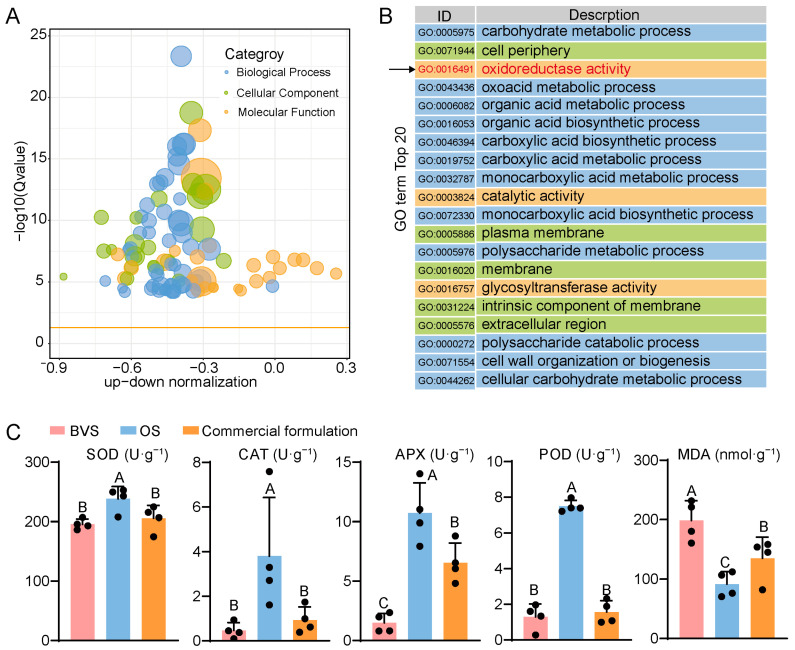
(**A**) Up-down normalization (**B**): The top 20 enriched GO terms for the DEGs in BVS vs. OS gerbera cut flowers. (**C**) Analysis of antioxidant enzyme activity and MDA content in gerbera cut flowers. The dots in the graph represent the number of repetitions. Different uppercase letters indicate that the differences between them are significant at the *p* < 0.01 level of significance.

Further, we find that the term ‘oxidoreductase activity’ was significantly enriched by transcriptome GO enrichment (Figure 7A,B). Compared with the control group, the expression levels of multiple oxidoreductase genes in the treatment group were significantly up-regulated, including catalase, superoxide dismutase, and glutathione peroxidase-related genes.

When cut flowers are placed in vases, antioxidant enzymes in the plant body work synergistically to combat reactive oxygen species (ROS). The activities of key antioxidant enzymes, including CAT, APX, SOD, and POD, are widely used as important indicators for evaluating the efficacy of freshness preservatives for cut flowers. In the gerbera cut flowers applied with OS for 6 days, we found that the activities of CAT, APX, SOD, and POD were significantly higher than those in both control groups. Conversely, the MDA content in the OS group was significantly lower compared to the other groups (Figure 7C). These results indicate that the antioxidant capacity of gerbera cut flowers treated with the OS was the highest among the tested groups, surpassing those treated with the basic vase solution or the commercial preservative. This enhanced antioxidant activity effectively prolonged the VL of the cut flowers and improved their resistance to oxidative stress.

Here, we tested various agents to prolong the VL of gerbera cut flowers, demonstrating the efficacy of COS, CA, and CaCl_2_. More detailed, the combination of 104 mg/L of COS, 93 mg/L of CaCl_2_, and 92 mg/L of CA used as a compound preservative formula for the fresh-keeping treatment of ‘Minghui Zixia’ can delay the senescence of its cut flowers.

## 4. Discussion

As a newly developed variety of gerbera cut flowers, ‘Minghui Zixia’ has great market potential in China. However, post-harvest preservation challenges have long hindered the growth of the gerbera cut flower industry. In this study, we developed an OS with the combination of an antimicrobial agent, organic acid, and inorganic salt, which significantly extends the VL of gerbera cut flowers. This advancement is of great importance for promoting the development and market competitiveness of the gerbera cut flower industry. Moreover, our findings revealed that the OS had a significant impact on antioxidant enzyme activity. Transcriptome analysis further demonstrated changes in the expression of genes involved in cytokinin synthesis and stress tolerance, suggesting that these factors play a key role in prolonging the VL of gerbera cut flowers treated with the OS. Our results confirmed the effectiveness of the new preservative formula, which holds great significance for the advancement and growth of the gerbera cut flower industry.

### 4.1. The Combination of COS, CA, and CaCl_2_ Can Significantly Extend the VL of Gerbera

Decreases in VL of cut flowers are primarily attributed to the extensive reproduction of microorganisms at the cut ends of flower stems [12]. Residues block the vessels of flower stems, and air enters the stems, causing the blockage of air plugs and xylem vessels and thereby reducing the flow and absorption of water. The water balance in the cut flower is broken, and then the phenomena of wilting and death occur [27,28]. The use of compound preservatives is also an important measure to extend the life of fresh-cut flowers [29]. To mitigate this issue, it is recommended to recut flower stems to remove embolisms and immediately place them in water; however, this must be conducted in clean water to prevent bacterial contamination, which can negate the benefits of recutting [30]. In addition, compared with the control group, the senescence of cut flowers of ‘Minghui Zixia’ in the treatment group showed significantly delayed senescence. The VL was extended by 7 days, and there was an increase in maximum flower diameter by 1.12 cm. These results are similar to those of research on delaying the senescence of *Rosa hybrida* [31] and *Eustoma grandiflorum* L. [32] by using composite preservative treatment. At the same time, COS, CA, and CaCl_2_ have been shown to delay the senescence of many cut flowers [33,34,35,36]. Compared with widely used commercial preservatives, our compound preservative formula significantly extends the VL of gerbera cut flowers, demonstrating strong commercial potential. These promising results also motivate further exploration of the underlying mechanisms by which this preservative delays senescence.

### 4.2. Composite Preservatives Improve the Lifespan of Gerbera by Upregulating Antioxidant Enzymes

The scavenging function of reactive oxygen species is closely related to the maintenance of plant drought tolerance [37]. During senescence, plants often experience an imbalance in ROS scavenging, leading to the toxic effects of ROS on cells [38]. Key antioxidant enzymes, such as POD, SOD, and CAT, play a crucial role in neutralizing free radicals and mitigating oxidative damage [39,40]. In this study, the CAT, APX, SOD, and POD activities of gerbera cut flowers in the treatment group were 3.8, 10.72, 238.49, and 7.5 U g^−1^, respectively, significantly higher than those in the control group. The MDA content of gerbera cut flowers was 91.5 nmol g^−1^, significantly lower than that of the control group. This indicates that the preservative solution effectively removed excessive ROS generated under stress and inhibited MDA accumulation. Furthermore, it significantly upregulated CAT, POD, and SOD activities in the petals, maintaining the balance of the antioxidant system. These results suggest that the upregulation of antioxidant-related enzyme activity is a key mechanism through which the compound preservative delays the senescence of gerbera cut flowers. Previous studies have similarly reported that ROS-scavenging enzyme activities can be significantly enhanced under external treatments, and this enhancement is closely associated with the extended lifespan of flowers [41,42,43]. Here, we preliminary elucidated how the compound preservative extends the VL of gerbera cut flowers through enhancing antioxidant enzyme activities.

### 4.3. Salicylic Acid and Cytokinin Signaling Activation Are Key Factors in Improving the Lifespan of Gerbera Cut Flowers

To further investigate the effects of the compound preservative developed in this study, we conducted transcriptomic analyses to explore its impact on gene expression in gerbera. The results revealed that the compound preservative treatment significantly activated genes associated with phytohormone signaling and pathogen defense. In particular, the cytokinin (CK) and salicylic acid (SA) signaling pathways were notably upregulated under the preservative treatment. These two plant growth regulators are closely linked to flower senescence [44]. Cytokinin signaling, in particular, plays a crucial role in plant anti-aging processes. Our findings showed that the compound preservative treatment significantly activated the transcription of genes involved in cytokinin synthesis and its signaling pathway. This increase in cytokinin production and the activation of its signaling pathways are known to enhance plant tolerance to abiotic stress and delay senescence [45,46].

Salicylic acid (SA) is a key signaling molecule in plant defense against pathogens [47]. Our results show that compound preservative treatment significantly enhanced the expression of key genes in the SA signaling pathway, including *GhTGA1*, *GhTGA4*, *GhNPR1*, and *GhRBOHA*. These genes play a central role in plant defense responses to pathogens, and the upregulation of their expression may enhance the resistance of gerbera cut flowers to potential pathogens such as bacteria. *TGA1* and *TGA4* bind to NPR1 proteins to activate defense-related genes such as *PR-1* [48]. They also include some wax-synthesizing genes. Wax, as the first line of defense on the plant surface, plays an important role in resisting environmental stress. *KCS* (3-ketoyl-CoA synthetase) is a rate-limiting enzyme in the biosynthesis of VLCFA (ultra-long-chain fatty acids), which is one of the main components of wax [49]. Studies have shown that the overexpression of the *KCS* gene can significantly increase the wax content on plant leaves and reduce the water evaporation rate, thus improving the drought tolerance of plants [50]. For cut flowers, reducing water evaporation is one of the key factors in extending their VL. The enhancement in the waxy layer reduces water loss and maintains the freshness and vitality of the flowers. For example, in one study, the VL of cut flowers was significantly extended by treating them with water containing hydrogen, possibly due to the fact that the hydrogen treatment promoted the formation of waxy layers and reduced water evaporation. In addition, genes associated with plant wax synthesis and stress response, such as *KCS5*, *CUT1*, and *KCS6*, were also significantly upregulated after treatment, which may help enhance the plant’s physical barrier and reduce water evaporation, thereby extending VL [51].

## 5. Conclusions

In conclusion, the compound preservative extends the VL of gerbera cut flowers by enhancing antioxidant enzyme activities, which might result from the activating of the cytokinin and salicylic acid signaling pathways. This combined effect may lead to a delay in the aging process of cut flowers, thus significantly extending the VL. These findings offer a novel strategy for preserving gerbera cut flowers and may also inspire advancements in the preservation of other cut flower varieties.

Our study not only provided the molecular mechanisms underlying the effectiveness of the compound preservative but also provided a scientific foundation for the development of new and more efficient techniques for cut flower preservation.

## Data Availability

The original contributions presented in the study are included in the article, and further inquiries can be directed to the corresponding authors.

## Supplementary Materials

The following supporting information can be downloaded at https://www.mdpi.com/article/10.3390/biology14010018/s1. Table S1: Box–Behnken horizontal test design and results. Table S2: Different preservative formulations of gerbera cut flower. Table S3: Gene-specific primers. Table S4: Analysis of variance of regression equation. Table S5: Model feasibility analysis. Table S6: Quality of RNA-Seq data from petals of a gerbera cut flower.
